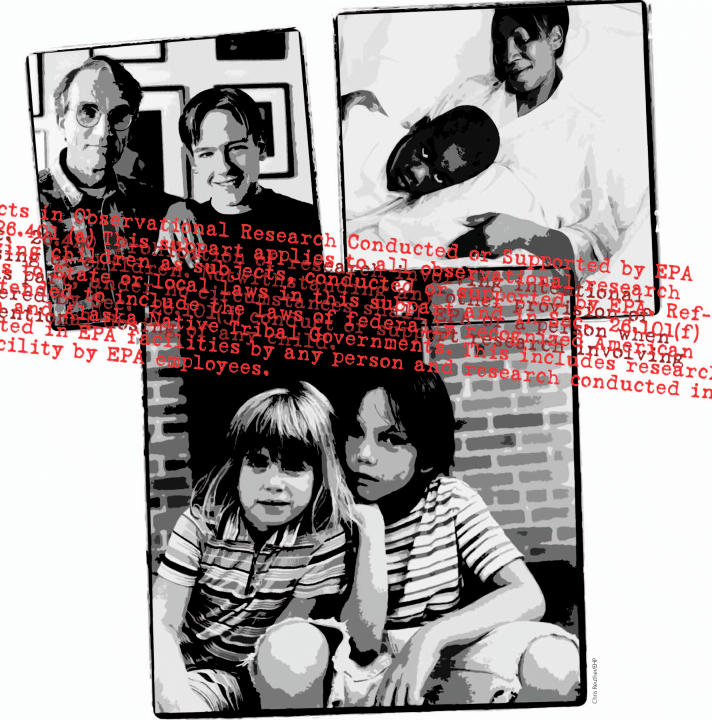# Human Experimentation: A Rule Gone Awry?

**DOI:** 10.1289/ehp.114-a360

**Published:** 2006-06

**Authors:** Adrian Burton

The U.S. EPA’s new Protections for Subjects in Human Research rule, which came into force on 7 April 2006, was born of a need to tighten the ethical guidelines controlling nonmedical human experimentation. The rule was ostensibly designed to offer people greater protection in pesticide toxicity experiments. But just two weeks after its coming into force, a coalition of labor and environmental interest groups filed suit against the EPA, challenging the rule’s legality and ethics. Against a backdrop of claims of industry influence, financial interests, and bipartisan rhetoric, the Second Circuit Court of Appeals in New York City must now determine whether this rule safeguards Americans against unethical experimentation or sells them out to big business.

The plaintiffs—the Natural Resources Defense Council (NRDC), Pesticide Action Network North America, San Francisco Bay Area Physicians for Social Responsibility, and Northwest Treeplanters and Farmworkers United, Oregon’s union of farm, nursery, and reforestation workers—filed their suit on 23 February 2006. They claim the new rule does not meet the demands of Congress to afford the fullest protection to human subjects—especially pregnant women and children—in pesticide experiments, and charge that the rule is undercut by numerous loopholes that ultimately encourage, rather than deter, human testing.

“EPA is giving its official blessing for pesticide companies to use pregnant women, infants, and children as lab rats in flagrant violation of [the EPA Appropriations Act of August 2005] cracking down on this repugnant practice,” said Erik Olson, senior attorney for the NRDC, in a 23 January 2006 press release from that organization. “There is simply no legal or moral justification for the agency to allow human testing of these dangerous chemicals. None.”

## The Need for a New Rule

The new rule expands on the existing Federal Policy for the Protection of Human Subjects (or “Common Rule”), which covers the ethics of medical trials and governs human research sponsored or regulated by federal agencies. The Common Rule largely reflects the aims of the Nuremberg Code, a document drawn up after World War II providing the basis for modern human experimentation ethics. But according to *Human Pesticide Experiments*, a June 2005 report drawn up for Senator Barbara Boxer (D–CA) and Representative Henry Waxman (D–CA), the Common Rule offers insufficient protection against pesticide companies that pay people to be intentionally exposed to their products.

Why would a pesticide company even want to conduct experiments on humans in the first place? One key reason can be found in the provisions of the 1996 Food Quality Protection Act, which was passed to provide greater protection for vulnerable populations (such as children) against pesticide exposures via food. The act applied a 10-fold safety factor to permissible levels of pesticide residues in food to account for children’s greater vulnerability. Under the act, pesticides could be granted a lower safety factor “only if, on the basis of reliable data . . . [the lower factor] will be safe for infants and children.” According to a 2004 National Academy of Sciences report titled *Intentional Human Dosing Studies for EPA Regulatory Purposes: Scientific and Ethical Issues*, several pesticide manufacturers conducted human dosing studies in pursuit of lower safety factors.

But human testing raises important ethical questions that need to be answered. For example, although the subjects in these pesticide dosing studies were supposed to have given their consent to participate, was it ethical to have asked them in the first place? Would the benefits of these studies outweigh the risks to the subjects (as recommended by the 1964 Declaration of Helsinki on human medical experimentation), or just help the bottom line of the companies involved?

Given the dilemma surrounding human experimentation, in 1998 the Clinton administration placed a moratorium on the EPA reviewing human experiments for setting permitted exposure levels. When the moratorium was lifted by the Bush administration after a court found procedural errors in its establishment, Congress reacted by way of the 2005 EPA Appropriations Act, demanding that the agency draw up new ethical guidelines governing itself and all third parties wishing to submit results to the agency for regulatory purposes. Championing this cause was Representative Hilda Solis (D–CA), along with Boxer and Waxman, whose 2005 report claimed that 22 human pesticide experiments submitted to the EPA for possible use in regulatory decision making were in violation of ethical and scientific standards, for reasons such as failure to obtain fully informed consent, dismissal of adverse outcomes, and the use of unethical liability waivers.

In obedience to Congress, the EPA drew up a proposed rule, which evolved into the final rule published on 2 February 2006 after a 90-day public comment period. During this time the EPA received thousands of criticisms that it took into account for preparing the final draft—a document that ended up little to the liking of the litigating coalition or indeed of the politicians who had demanded it.

The coalition members allege that the rule’s wording—with what they perceive to be inherent loopholes—now actually encourages rather than prohibits human experimentation, and suggest that pesticide companies could take advantage of this to further their interests. “EPA’s rule allows pesticide companies to use intentional tests on humans to justify weaker restrictions on pesticides,” said Margaret Reeves, a senior scientist and program coordinator with Pesticide Action Network North America, in a press release from that group announcing the filing of the lawsuit. In the same press release, Robert Gould, president of San Francisco Bay Area Physicians for Social Responsibility, was quoted as saying, “Pesticide companies should not be allowed to take advantage of vulnerable populations by enticing people to serve as human laboratory rats.”

In a press release issued by his office the same day, Waxman commented: “Unethical human pesticide experiments must be stopped. It is morally wrong to encourage chemical companies to dose humans with pesticides in order to argue for weaker public health standards.”

## Question of Intent

One of the major intentions for the new rule—and now a major bone of contention—was that it ban the experimental use of pregnant women and children. Indeed, the EPA insists the rule does just that. “This rule provides far-reaching protections for all Americans and absolute protections for children and expectant mothers,” explains senior policy advisor William Jordan of the EPA Office of Pesticide Programs. “It categorically inhibits EPA or any researcher from using [such subjects] in intentional dosing studies. The rule further extends those protections by banning any researcher for pesticides from using pregnant women or children as participants in any intentional dosing study intended for submission to EPA.”

Jordan says the rule also prohibits the EPA from relying on any intentional pesticide dosing study involving pregnant women or children regardless of the intent of those conducting the study or the country where the study was conducted. Finally, he says, the rule directs the EPA to waive that prohibition “only if the agency were to become aware of information that would indicate the need for stricter regulatory controls for a pesticide.”

The coalition, however, points to what it considers to be several exceptions to the rule. They note that while Americans may be offered some protection from intentional dosing studies, the rule does nothing to prevent U.S. pesticide companies from performing experiments on nationals in other countries.

The term “intended for submission to the EPA” worries them too. “The wording of the rule means you are not allowed to do a study on pregnant women or children that you admit was intended from the beginning for submission to the EPA,” explains Olson. “If that was not your original idea—or if you say it wasn’t—then you apparently could do those experiments. A second scenario could be where a company performs a study on infants or pregnant women and submits it to a state or, say, a European country, saying that it does not intend to submit it to the EPA. We have plenty of experience to show that decisions made by other bodies are very influential on the EPA. [So], you can avoid the EPA rule and still get the result you want.” In addition, pesticide studies on pregnant women and children submitted under clean water, drinking water, clean air, hazardous waste, or other laws are not covered by the new rule’s restrictions, Olson explains.

Jordan rejoins that the rule does not, in fact, permit a registrant to claim at the outset of a study that they have no intent to submit a human pesticide study and then later submit that study. “If the agency were ever to receive such a study done in this deceptive fashion,” he insists, “the rule prevents EPA from using it.”

## Children’s Consent

Another major area of contention is consent. In the proposed rule, section 26.408 of the text clearly stated that “if the [institutional review board] determines a research protocol is designed for conditions or for a subject population for which parental or guardian permission is not a reasonable requirement to protect the subjects (for example, neglected or abused children), it may waive the consent requirements in subpart A of this part and paragraph (b) of this section.” Subpart A refers to the requirement that a child must assent to be included in an experiment (although this was apparently not necessary “if the capability of some or all of the children is so limited that they cannot reasonably be consulted”), while paragraph (b) refers to soliciting permission from parents or guardians. Many critics believed this waiver suggested that abused or mentally impaired children could be freely used in commercial pesticide experiments.

In comments published with the final rule, the EPA says such a sinister reading is incorrect: “Many commenters misinterpreted EPA’s proposed language. Contrary to public comments, none of the alleged ‘loopholes’ ever existed, because the prohibition in proposed Sec. 26.420 stated ‘Notwithstanding any other provision of this part, under no circumstances shall EPA or a person when covered by Sec. 26.101(j) conduct or support research involving intentional dosing of any child.’”

According to the EPA, the words “Notwithstanding any other provision of this part” meant that the provisions in proposed Section 26.420 overrode all other provisions of the entire regulation, including those in 26.408. So even though the latter section would have appeared to give the EPA the authority to waive certain requirements, it did not, the agency claims, authorize any departure from the ban declared in Section 26.420. In Jordan’s words, no child is going to be used in intentional dosing studies—period.

“But if this is what it means, why doesn’t it simply say that?” asks Olson. “It sure appears that if there is no ‘intent to submit’ to EPA then you could use such children.”

The wording in the final rule is scarcely different from that in the original version. The proposed Section 26.408, now named Section 26.406, remains virtually intact, while the promise in the proposed Section 26.420 has been consolidated into a blanket statement in the final Section 26.203 that “under no circumstances shall EPA conduct or support research involving intentional exposure of any human subject who is a pregnant woman (and therefore her fetus) or child.”

Other concerns voiced by the coalition include claims that the ethics review board established by the new rule is powerless to prevent experiments it deems unethical (its role is merely advisory), that nowhere is any sanctioning power mentioned, and that a clause in the text requires that any studies presented need only “substantially” comply with the rule—a quantity of compliance that is never defined.

Given the EPA’s funding of the now-cancelled CHEERS study (which would have paid parents to let EPA and industry scientists observe the effects on their children of spraying their homes with pesticide, but which was abandoned by the agency in the face of overwhelming criticism; the EPA declined to comment to *EHP* on the study), the fundamental question arises as to whether the agency should be allowed to write its own rules. It is now the job of the courts to decide whether the EPA has done a good job. Briefings will begin 5 June 2006, but a ruling could take a year or more to come through. In the meantime, the new rule is in force.

## Figures and Tables

**Figure:**